# Study on Colloidal Model of Petroleum Residues through the Attraction Potential between Colloids

**DOI:** 10.1155/2016/4182164

**Published:** 2016-04-19

**Authors:** Long-li Zhang, Guo-hua Yang, Chao-he Yang, Guo-he Que

**Affiliations:** ^1^College of Science, China University of Petroleum (East China), Qingdao, Shandong 266555, China; ^2^State Key Laboratory of Heavy Oil Processing, China University of Petroleum (East China), Qingdao, Shandong 266555, China

## Abstract

The samples of DaGang atmospheric residue (DG-AR), Middle East atmospheric residue (ME-AR), TaHe atmospheric residue (TH-AR), and their thermal reaction samples were chosen for study. All the samples were fractioned into six components separately, including saturates plus light aromatics, heavy aromatics, light resins, middle resins, heavy resins, and asphaltenes. The dielectric permittivity of the solutions of these components was measured, and the dielectric permittivity values of the components can be determined by extrapolation, which increased steadily from saturates plus light aromatics to asphaltenes. Moreover, the Hamaker constants of the components were calculated from their dielectric permittivity values. The Van der Waals attractive potential energy between colloids corresponding to various models could be calculated from the fractional composition and the Hamaker constants of every component. It was assumed that the cores of colloidal particles were formed by asphaltenes and heavy resins mainly; the other fractions acted as dispersion medium. For the three serials of thermal reaction samples, the Van der Waals attraction potential energy between colloids for this kind of model was calculated. For TH-AR thermal reaction samples, the Van der Waals attraction potential energy presented the maximum as thermal reaction is going on, which was near to the end of coke induction period.

## 1. Introduction

Petroleum residues are found to be colloidal systems, and asphaltenes are considered to be the cores of them [1–3]. Asphaltenes, which are the heaviest and the most polar fraction in residue, play a key role in the processing of heavy oil. The destruction of colloidal stability of heavy oil induces asphaltene precipitation and causes troubles to many petroleum processes including production, transportation, or refining.

Since the colloidal stability plays an important role in the usage of heavy oil, the colloidal model and colloidal stability of heavy oil have been widely studied. Because petroleum residue is a kind of complicated mixture, composed of innumerable molecules, it has to be divided into fractions based on polarity and solubility difference for further study [4–6]. Based on the solubility difference and chromatography separation, the former researchers found that the polarity of residue fractions increased steadily as the following sequence: saturates plus light aromatics, heavy aromatics, light resins, middle resins, heavy resins, and asphaltenes [7, 8]. The fractions distribution of residue played an important role in the colloidal stability, the reaction characteristics, and the emulsification performance of residue [9].

Although many researchers have presented various micelle models for residue, there are no models based on the direct potential energy between colloids derived from the composition and characteristics of fractions. Moreover, the colloidal stability is influenced by the fractions distribution and the characteristics of heavy oil [10–13]. The mean dipole moment of the fractions can be measured [12]. For example, the researchers pointed out that SARA composition influenced the stability of asphaltenes in heavy oil [14–16]. But SARA composition partition is blurry, based on the solubility and polarity of molecules, and every fraction was a complicated mixture. Although the contents and the characteristic of SARA fractions determine the colloidal stability of heavy oil together, the relationship between them cannot be forecasted integrally and quantitatively.

Some researchers pointed out that resins were helpful to the stability of asphaltenes [15, 16], so the increase of resin content would enhance the colloidal stability of heavy oil, and the increase of asphaltenes content would decrease the colloidal stability. It should be noted that resins are not required in order for asphaltenes to form stable nanocolloidal suspensions in toluene. As the division of asphaltene and resin is based on solubility, the characteristics of resin are adjacent to that of asphaltenes, and the characteristics of them are continuous. If the properties of the molecules are just on the critical point of asphaltenes and resins, it is difficult to deduce the effects of these molecules on the colloidal stability of residues, since this statement is qualitative and does not integrate the effect of properties and the composition of fractions of residue at the same time.

According to “the modified Yen model” proposed by Mullins and coresearchers [14], the asphaltene colloidal particles in crude oils were nanocolloidals, which were mainly composed of asphaltenes and the heaviest resins. The asphaltenes were in hierarchical structures in crude oils, such as molecules, nanoaggregates, and clusters of nanoaggregates. This work will study the attractive energy between the clusters dispersed in the medium of other fractions.

The colloidal stability of heavy oil is affected by the attractive potential and repulsive potential between colloid groups. This paper is to instruct the colloidal model of residue based on the attractive potentials between colloids for the sake of viewing the fractions distribution and fraction characteristics. This model will be used to reveal the colloidal stability variation during thermal reactions.

## 2. Experimental Section

There are three kinds of atmosphere residues studied, the boiling point of which was more than 350°C. The samples were DaGang atmospheric residue (DG-AR), Middle East atmospheric residue (ME-AR), and TaHe atmospheric residue (TH-AR). Their properties have been presented in the previous papers [8, 12].

The samples were upgraded to study the colloidal stability variation of residues during thermal reaction. Thermal reaction was performed under nitrogen and the initial pressure is 1.0 MPa at room temperature. The coke induction period of thermal reaction was 80 min for DG-AR at 405°C, 100 min for ME-AR at 400°C [12], and 60 min for TH-AR at 400°C. The heavy oil was separated into n-pentane asphaltene and n-pentane maltene. Then, the maltene was separated into fractions through liquid chromatographic separation; and the neutron aluminum oxide was used as adsorbent, which was pretreated and contained 5% wt. water [13]. The eluting solvents used were n-heptane, mixture of n-heptane and benzene (v : v = 85 : 15), mixture of n-heptane and benzene (v : v = 1 : 1), benzene, or mixture of benzene and ethanol (v : v = 1 : 1). The corresponding fractions could be gained as the following: saturates plus light aromatics, heavy aromatics, light resins, middle resins, and heavy resins. The colloidal stability variation of residue came from the shift of fractions distribution and the molecular characteristics of every fraction [8, 12].

The separation scheme, mean molecular weight, and the dielectric permittivity measuring methods were the same as described previously [8, 12, 13].

The fractions were diluted by benzene separately. Firstly, a stock solution of fraction was prepared. Then, the stock solution was distributed in vials, and benzene was added to each vial to achieve the preconcerted weight percentage. The solution was placed at least for 24 hours before measurement to ensure stabilization. The dielectric permittivity of the solutions of fractions was measured, and the dielectric permittivity of fractions can be gained when extrapolated to the 100%. The frequency used for permittivity measurements was 1 kHz. The Hamaker constants of the fractions were calculated from the dielectric characteristics of them. When the dielectric permittivity value is little, there are not notable differences between the long-range and the short-range Hamaker constant. The dielectric permittivity values of the components of petroleum residue were lower than 10. So the short-range Hamaker constant was used in this study. When the two particles were the same and existed in vacuum, the short-range Hamaker constant can be calculated as [19]

(1) A113264πhω1ε1−12ε1+13/2=3232hcλ1ε1−12ε1+13/2=ASM

*ε*_1_ is the extrapolated relative dielectric permittivity of certain fraction; *ω*_1_ is the characteristic absorption frequency; *λ*_1_is the characteristic absorption wavelength, nm; *h* is the Planck constant, 6.626176 × 10^−34^ J·s; *c* is the velocity of light, 3.0 × 10^8^ m·s^−1^.

Since the fractions of petroleum residue were complex mixture, the UV-vis absorption spectrum of them was wideband from 200 nm to 400 nm without specific fine structures [20], the characteristic absorption wavelength for fractions of petroleum residue was chosen as 300 nm. The Hamaker constants of fractions could be computed by (1). The researchers have proposed various colloidal model of residue [14, 17, 18]. The Van der Waals attraction potential energy of different model could be calculated from the fractional composition and the Hamaker constants of every component.

## 3. Results and Discussions

### 3.1. The Dielectric Permittivity of the Solutions of Fractions

**Figure 1:**
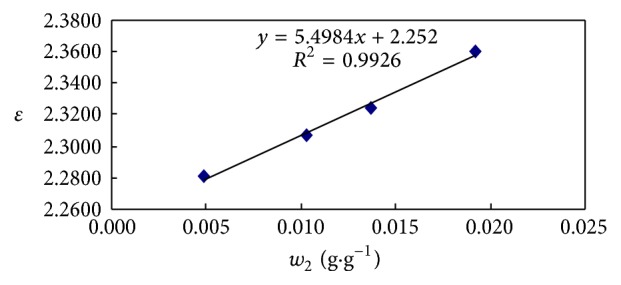
The dielectric permittivity values as a function of concentration for solution of asphaltenes of TH-AR thermal reaction sample for 40 min.

The dielectric permittivity of the solutions of fractions was measured. The asphaltene of TH-AR thermal reaction sample for 40 minutes was illustrated, as exampled. It could be seen from Figure 1 that the dielectric permittivity of solution increased with concentration, and the value for the neat material can be determined by extrapolation to 100%, which was 7.75. The dielectric permittivity of other samples could be gained also and shown in Tables 1 [Table tab2]–3.

It can be seen in Tables 1–3 that the dielectric permittivity values increased following the sequence, saturates plus light aromatics, heavy aromatics, light resins, middle resins, heavy resins, and asphaltenes, which was consistent with the polarity variation of them. Furthermore, all dielectric permittivity values were lower than 10, much lower compared to water which was 78. From the low dielectric permittivity values determined, the Hamaker constants of fractions could be calculated by (1) [19].

### 3.2. The Van Der Waals Attractive Potential Energy between Colloids for Different Model

The Van der Waals attractive potential energy between colloids for different models can be calculated by Hamaker constant of the fractions and the fractions distribution. The colloidal model with the lowest energy will be the most stable construction in view of attractive energy. Seven kinds of models would be put forward for comparison.

**Figure 2:**
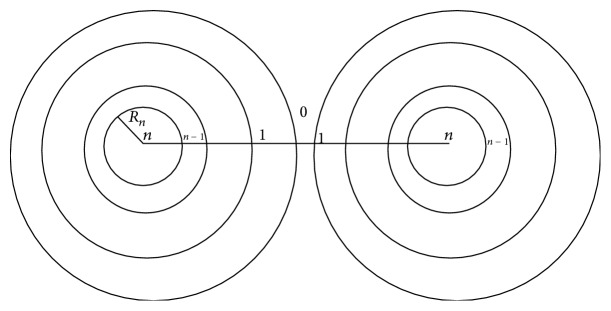
The diagram of two particles with adsorbed layers.

Vold deduced the effect of solvation sheaths on the Van der Waals attraction between spherical colloid particles [21], which can be calculated as (2)–(5) and were shown in Figure 2 [21–23]:

(2) −12V∑i,j=1,naiajHij,

(3) aiAi1/2−Ai−11/22,

(4) HijHji=Hδij2Rk,RlRk,

(5) Hx,yyx2+xy+x+yx2+xy+x+y+2ln⁡x2+xy+xx2+xy+x+y.

The subscript zero was assigned to the medium and successive layers of adsorbed material are numbered from the outside inward as 1, 2,…, *n* − 1; the unsolvated particle is numbered *n*.

*A*_*i*_ is the Hamaker constant of the shell numbered *i*.

*R*_*k*_ is the radius of the shell *i* or *j*, which has the smaller radius, *R*_*l*_ is the other, and *δ*_*ij*_ is the separation of the surface of the two spheres. *H* is the functor defined by (4) and (5).

The seven kinds of models were presented in Table 4. The asphaltenes, which were the largest and most aromatic molecules, were in hierarchical nanocolloidal structures in heavy oils.

For the model #2, when the radius of asphaltene core was considered 10 nm, the heavy resin adsorbed layer thickness can be computed based on the relative content of them. Then, the thickness of other adsorbed layers could be computed. The distance between the outer surfaces is fixed as 0.3 nm [22]. The attractive energy potential can be computed from the Hamaker constants and the composition of fractions for certain sample, at certain model, and for certain radius of cores.

**Figure 3:**
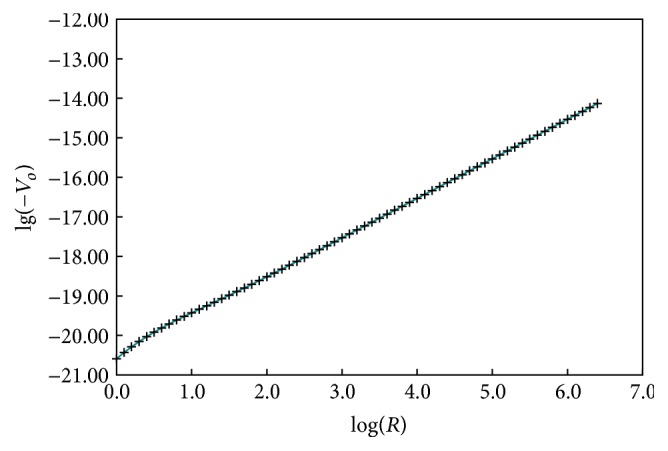
The variation of attractive potential energy with radius values of TH-AR for model #7.

As the radius of asphaltene cores varied from 1.0 nm to larger values, the attractive potential energy at different radius values for TH-AR of model #7 could be calculated, which was shown in Figure 3.

**Figure 4:**
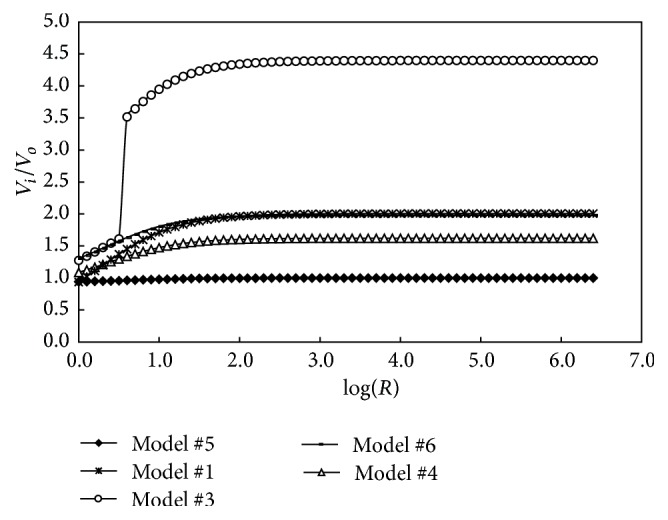
The ratios of the attractive potential energy for different model to that of model #7 for DG-AR.

It can be seen in Figure 3 that the attractive potential energy increased with the increase of radius of asphaltene cores. To compare the attractive potential energy of different model, the ratios of the attractive potential energy of different model to that of model #7 were calculated at different colloidal radius and shown in Figure 4.

It can be seen in Figure 4 that, for DG-AR, the energy for model #5 approximately equals to 1, and the others were more than 1. So model #5 and model #7 were the most possible models for DG-AR in view of attractive potential energy. Most of the other studied samples presented the same phenomenon.

Furthermore, all the samples were studied and the result showed that the model #2 was the most stable model for almost all the samples, except for two thermal reaction products from ME-AR.

The modified Yen model is confirmed and widely accepted [14]; the attractive potential energy under model #7 was calculated and shown in Tables 5–7, assuming that the asphaltene radius equaling 10 nm or 50 nm. The colloidal stability parameters of the residual samples were characterized through mass fraction normalized conductivity method [12]. The coke induction period of thermal reaction was 80 min for DG-AR, 100 min for ME-AR, and 60 min for TH-AR.

It can be seen from Tables 5–7 that for the studied samples, as thermal reaction is going on, the colloidal stability parameters decreased steadily. It can be seen from Table 5 that for the TH-AR thermal reaction samples, when radii are equaling 50 nm, the attractive potential energy between colloids increased as thermal reaction is going on, which was consistent with the colloidal stability parameters decreasing. For the TH-AR thermal reaction samples, when radii are equaling 10 nm, the attractive potential energy got to the maximum at 80 minutes, which was almost the same to the end of coke induction period and decreased after that, as thermal reaction is going on. The coke induction period was defined to be the time at which 0.1 wt% coke was produced.

It can be seen from Table 6 that, for the DG-AR thermal reaction samples, the attractive potential energy presented the maximum at 120 minutes, except for the DG-AR, no matter whether radii are equaling 10 nm or 50 nm. It can be seen from Table 7 that, for the ME-AR thermal reaction samples, the attractive potential energy presented an increasing tendency except for the ME-AR, no matter whether radii are equaling 10 nm or 50 nm. The possible explanation is that the colloidal stability is determined by the attractive energy and the repulsive energy.

Finally, the repulsive potential energy was studied. The Zeta potentials of the solutions of ME-AR and thermal reaction samples were measured by a Phase Analysis Light Scattering Potential Analyzer. The electric repulsive potential energy between residue colloids was computed and shown in Table 8 [24].

It can be seen in Table 8 that the dielectric repulsive potential energy between residue colloids was far less than that of attractive potential energy, which was about one tenth of the latter. The attractive potential energy values were determined supposing that the radii were 50 nm, and the expected attractive potential energy values would be bigger as the radii were larger than 50 nm. Perhaps the stabilization effect mainly came from the steric repulsive strength from alkyl chain of the asphaltenes and resins. So the key factors for colloidal stability of residue are Van der Waals attractive potential energy and steric repulsive potential energy.

## 4. Conclusion

Firstly, the results showed that the modified Yen model proposed by Mullins provided lower attractive potential energy for most residues.

Secondly, for TH-AR thermal reaction samples when radii of cores are equaling 50 nm and for ME-AR thermal reaction thermal samples except for ME-AR, the Van der Waals attractive potential energy increased steadily as thermal reaction is going on, which was consistent with the colloidal stability parameter decreasing. These phenomena showed that the attractive potential energy played a key role in the colloidal stability of residue.

Thirdly, the dielectric repulsive potential energy was far less than the Van der Waals attractive potential energy between residual colloids, indicating that the key factors for colloidal stability of residue are Van der Waals attractive potential energy and steric repulsive potential energy.

## Figures and Tables

**Table 1:** Dielectric permittivity of samples of DG-AR thermal reaction at 405°C.

Reaction time/min	Saturates plus light aromatics	Heavy aromatics	Light resins	Middle resins	Heavy resins	Asphaltenes
DG-AR	2.52	3.49	3.48	4.79	5.76	7.38
40	2.29	3.04	3.41	4.01	5.38	6.65
80	2.40	3.07	3.18	3.61	5.19	7.99
120	2.40	3.10	3.27	4.16	5.23	10.20
160	2.44	2.80	3.56	4.00	5.30	9.13

**Table 2 tab2:** Dielectric permittivity of samples of ME-AR thermal reaction at 400°C.

Reaction time/min	Saturates plus light aromatics	Heavy aromatics	Light resins	Middle resins	Heavy resins	Asphaltenes
ME-AR	2.50	2.94	3.79	4.68	5.30	9.21
40	2.58	3.43	3.77	3.57	4.84	8.83
80	2.46	3.14	4.11	4.89	5.17	6.86
100	2.50	4.76	4.68	5.65	5.49	7.39
160	2.47	3.09	3.97	5.54	5.99	7.56

**Table 3:** Dielectric permittivity of samples of TH-AR thermal reaction at 400°C.

Reaction time/min	Saturates plus light aromatics	Heavy aromatics	Light resins	Middle resins	Heavy resins	Asphaltenes
TH-AR	2.46	2.85	3.24	4.30	5.51	6.79
40	2.50	3.06	3.91	4.36	6.22	7.75
80	2.39	3.58	4.19	4.30	5.08	8.53
120	2.54	3.85	4.13	5.55	4.99	6.32
160	2.34	3.35	4.06	5.13	5.51	5.37

**Table 4:** The suggested colloidal model.

	Cores	Absorbent	Medium	Proposed by
Model #1	Asphaltenes		Heavy resins, middle resins, light resins, heavy aromatics, and saturates plus light aromatics	Mack [17]
Mode #2	Asphaltenes	Heavy resins, middle resins, light resins, and heavy aromatics, steadily	Saturates plus light aromatics	Pfeiffer and Van Doormaal [18]
Mode #3	Asphaltenes and heavy resins		Heavy resins, middle resins, light resins, heavy aromatics, and saturates plus light aromatics	
Mode #4	Asphaltenes	Heavy resins	Middle resins, light resins, heavy aromatics, and saturates plus light aromatics	
Mode #5	Asphaltenes	Heavy resins and middle resins	Light resins, heavy aromatics, and saturates plus light aromatics	
Mode #6	Asphaltenes, heavy resins, and middle resins		Light resins, heavy aromatics, and saturates plus light aromatics	
Mode #7	Asphaltenes and heavy resins	Middle resins	Light resins, heavy aromatics, and saturates plus light aromatics	Mullins [14]

**Table 5:** The attractive energy between colloids of TH-AR for model #7.

Reaction time/min	The energy when *R* = 10 nm (J)	The energy when *R* = 50 nm (J)	Colloidal stability parameters
0 (TH-AR)	−3.74 × 10^−20^	−1.58 × 10^−19^	1.45
40	−4.45 × 10^−20^	−1.60 × 10^−19^	0.82
80	−5.01 × 10^−20^	−1.72 × 10^−19^	0.38
120	−4.79 × 10^−20^	−2.59 × 10^−19^	0.28
160	−4.87 × 10^−20^	−2.72 × 10^−19^	0.20

**Table 6:** The attractive energy between colloids of DG-AR for model #7.

Reaction time/min	The energy when *R* = 10 nm (J)	The energy when *R* = 50 nm (J)	Colloidal stability parameters
DG-AR	−5.45 × 10^−20^	−2.86 × 10^−19^	8.20
40	−3.65 × 10^−20^	−1.70 × 10^−19^	2.29
80	−2.65 × 10^−20^	−9.68 × 10^−20^	1.14
120	−4.48 × 10^−20^	−1.78 × 10^−19^	0.67
160	−3.65 × 10^−21^	−1.44 × 10^−19^	0.71

**Table 7:** The attractive energy between colloids of ME-AR for model #7.

Reaction time/min	The energy when *R* = 10 nm (J)	The energy when *R* = 50 nm (J)	Colloidal stability parameters
ME-AR	−5.18 × 10^−20^	−2.49 × 10^−19^	1.70
40	−2.05 × 10^−20^	−6.31 × 10^−20^	1.22
80	−4.80 × 10^−20^	−2.42 × 10^−19^	1.04
100	−6.07 × 10^−20^	−3.19 × 10^−19^	0.86
160	−6.76 × 10^−20^	−3.52 × 10^−19^	0.60

**Table 8:** The electric repulsive potential energy between colloids.

Sample	*ξ* (mV)	*R* (nm)	*U*_*R*_ (J)	Attractive potential energy (*R* = 50 nm)
0 (ME-AR)	−41.77	55 ± 1.25	1.06 × 10^−21^	−2.49 × 10^−19^
ME-AR, 40 min	26.91	145 ± 1.25	1.16 × 10^−21^	−6.31 × 10^−20^
ME-AR, 100 min	31.79	180 ± 1.25	2.01 × 10^−21^	−3.19 × 10^−19^
